# Gridded daily rainfall data for Ghana for the period 1960 - 2015: Approach and validation process

**DOI:** 10.1016/j.dib.2023.109115

**Published:** 2023-04-05

**Authors:** Steve Ampofo, Thompson Annor, Jeffrey N.A. Aryee, Jacob Agyekum, Leonard K. Amekudzi

**Affiliations:** a Department of Meteorology and Climate Science, Kwame Nkrumah University of Science and Technology, Kumasi, Ghana; b Department of Environmental Science, School of Environment and Life Sciences, C. K. Tedam University of Technology and Applied Science (UTAS), Navrongo, Ghana

**Keywords:** Daily rainfall, Interpolation, Minimum surface curvature, High resolution, Point-pixel, Gridding, Ghana

## Abstract

Rainfall data is necessary at resolutions that allow modelling of environmental change and its impact on socio-economic well-being. This is particularly so with agricultural output determination in much of Africa with Ghana not exemption, where data is required for intra and inter-seasonal assessment of the impact of rainfall on yield. However, the sparse and limited distribution data from gauge measurements, coupled with periods of no record of daily rainfall data, limit their application for any meaningful endeavour. This data cures such deficiency by generating a high resolution (0.25° × 0.25°) gridded daily rainfall using the Minimum Surface Curvature (MSC) interpolation for 190 stations distributed across all agro-climatic zones of Ghana. Validation is done using 19 Ghana Meteorological Agency (GMet) gauge stations (10%) by comparing the; ratios, correlations and Root Mean Square Error (RMSE) of the observed to the gridded for the seasons.

**Specifications Table**

**Table:** 

Subject	Environmental Science Climatology
Specific subject area	Meteorology (Daily Rainfall)
Type of data	Daily rainfall dataset for the period 1960 to 2015 covering the entire country Ghana (Netcdf file)
How the data were acquired	A total of 190 stations across the agro-climatic zones of Ghana (Table 2) were utilised as the input data. The distribution of the stations over the country is depicted in Fig. 2 and the corresponding number of stations for each zone is outlined in Table 2. The record for these stations were not complete at all times as there were inherent gaps for some days and months for some stations. This challenge was adequately addressed by the interpolation. A 56-year time frame (1960 - 2015) of daily rainfall data for each station results in 20,496 time-steps per station across the entire country. Data homogenisation was done using RHtestsV4 to determine stations with excessive change points which were excluded subsequently [1,2]. As detailed in [1], we performed the homogenisation test to identify change points (Fig. 1). Thereafter, we adjusted the past records, using the magnitude of change points as adjustment factors. Also, as a way of quality controlling the data, spurious records were manually detected and removed from the time series, since their inclusion in the spatial data gridding could produce wrong records and some ‘unrealistic’ inflections. The Minimum Surface Curvature (MSC) with tensioning interpolation technique was used to grid the data at a spatial resolution of 0.25^o^ × 0.25^o^. Thereafter, the data was processed into a netcdf file, followed by a point-pixel validation of the gridded dataset with data from unused stations. The Validation involved the use of 19 Ghana Meteorological Authority (GMet) gauge stations (10%) by comparing its Ratios, Correlations and Root Mean Square Errors to that of the gridded data to show the level of accuracy for the four (4) seasons (SON [Fall], DJF [Winter], MAM [Spring], JJA [Summer]. The Minimum Surface Curvature (MSC) interpolation method which is the application of mathematical interpolation concept based purely on Partial Differential Equation (PDE) was used [1,4]. It is universally applied where smooth approximation and interpolation is carried out for temperature, rainfall, water heads, positional data and potential fields. The MSC has universal application noted for its speed of computation for even large number of points but however is constrained by its complicated algorithm and limited ability to conserve extrapolation trends. Data homogenisation was done using RHtestsV4 to determine stations with excessive change points which were excluded subsequently [1,2]. Application of the Minimum Surface Curvature (MSC) interpolation at a 0.25° × 0.25° gridding resulted in a final netcdf file of daily rainfall dataset and the final output was validated using a point- pixel assessment [3]. The Validation involved the use of 19 GMet gauge stations (10%) by comparing its Ratios, Correlations and Root Mean Square Errors to that of the gridded data to show the level of accuracy for the four (4) seasons (SON [Fall], DJF [Winter], MAM [Spring], JJA [Summer]) and the performance of the interpolation technique for each of the 19 gauge stations used for the validation.
Data format	Input data: Excel CSV file for 190 stations Analyzed: NetCDF file (.nc)
Description of data collection	The output dataset is the results of an MSC with tensioning interpolation of 190 gauge station data obtained from the Ghana Meteorological Agency (GMet) across all the four (4) agro-ecological zones. This constituted the input data for which a daily data set for the 56 year period was generated which compensates for the lost data sets for some periods and some gauge stations under the period of study.
Data source location	Fig. 2 below shows the location of all gauge stations daily rainfall data used for the interpolation and Table 2 gives a summary of the distribution of the stations in all the four agro-ecological zones.
Data accessibility	Output Data: Repository name: Mendeley Data Data identification number: 10.17632/rmv8jh9thj.1 Direct URL to data: https://data.mendeley.com/datasets/rmv8jh9thj/1 Instructions for accessing these data: The dataset is provided here in a netcdf file (.nc) format. Input Data: Repository name: Mendeley Data Data identification number: 10.17632/rmv8jh9thj.2 Direct URL to data: https://data.mendeley.com/datasets/rmv8jh9thj/2 Instructions for accessing these data: The input dataset is a list of raw Excel Binary file for the 190 gauge stations. The periods of defective gauge readings or no readings are designated -999
Related research article	Not applicable

## Value of the Data

• Daily rainfall data necessary at resolution 0.25° × 0.25° allows the modelling of environmental change and its impact on socio-economic well-being, particularly with agricultural output.
• This dataset addresses the sparse and limited distribution of number of point data from -gauge measurements, coupled with periods of norecord of daily rainfall data in Ghana, which limits their application for any meaningful endeavour.
• The data is useful for all scientific inquiry requiring data that are specifically generated for the country Ghana as opposed to globally generated datasets which may suffer from the lack of local situational factors and limitations of input data sources [7].
• The data is relevant for crop models that require daily rainfall data for a tropical zone as it can be used to compute varied agro-climatological indices.
• Further use of this data may involve assessing the accuracy of the output data file from new validation or accuracy assessment methods or running same input data with a different interpolation method and making the right comparison.
• Meteorologist, Agricultural extension officers, Ecologist, Climate scientist, Forestry officials and hydrologist may find this daily rainfall data a much more enhanced and reliable data than gauge data in much of Africa which suffers inconsistencies.
• The dataset is very relevant for use by geographers, agriculturalist, foresters, meteorologist, climate scientist, etc. because of the inavailability of long-term daily rainfall data for most parts of the country. This is because, the distribution of gauge data is very sparse and there are mostly long periods of time when the equipment's may be defective and as such unreliable as a data source. This data set provides daily precipitation for every location (point/area) in Ghana, and can be accessed much easily, reducing the burden of researchers in having data for meteorological or climate studies.
• This data therefore cures, the data availability problem by ensuring an easy and wide dissemination of much needed dataset for researchers.
• Also, the dataset is much more reliable than other publicly available datasets for the reason that, it has a much longer record of about 56 years. Other available datasets have a very short span, which limits their usefulness for long-term application.
• The resolution of this dataset is much finer than global precipitation datasets which have very coarse resolution and have been produced without reference to local conditions.

## 1 Objective

The prime objective of this project was to develop a concise daily rainfall dataset spanning more than half a century and is reliable for climate studies, agro-meteorology, catchment hydrology and climatology. It provides, a comprehensive gridded data for the entire country, Ghana, at a high resolution of 0.25°. This means, daily rainfall data can be obtained for every single locality and point with known coordinates. This is very necessary as there are very few working Meteorological gauge stations which have very sparse distribution and very unreliable for research work in some localities [2].

## 2 Data Description

Two sets of data are provided in this work, the input data (Excel Binary) and an output NetCDF file which can be accessed with ARCGIS^Ⓡ^ software and other Programmes that runs on Linux, Python, etc.

The input data consist of 190 individual daily rainfall measurement from 190 stations distributed across all four (4) agro-ecological stations zones of Ghana (Fig. 2). This primary data was obtained from the Ghana Meteorological Authority (GMet). The list (Table 1) and distribution of the sourced primary data across all agro-ecological zones is provided in Table 2. These 190 point-data included agro-meteorological, climatological, Synoptic and experimental gauge stations. The 190 selected stations were selected based on a set threshold of not having more than 10% of missing data for the period 1960–2015. They were further sorted and rearranged into Year-Month-Day (YY-MM-DD) and the coordinates appended.

The selected stations were used for the Minimum Surface Curvature (MSC) interpolation [5] at a resolution of 0.25° × 0.25°. A point-pixel validation was undertaken to assess the accuracy of the final output file, which has daily rainfall measurement for all of the country Ghana for the 56-year study period. Examples of the utilisation of the final output data is the use to compute and display the number of wet and dry days for every point and locality of Ghana (Fig. 3) and the number of Wet spells (Fig. 4).

**Fig. 1:**
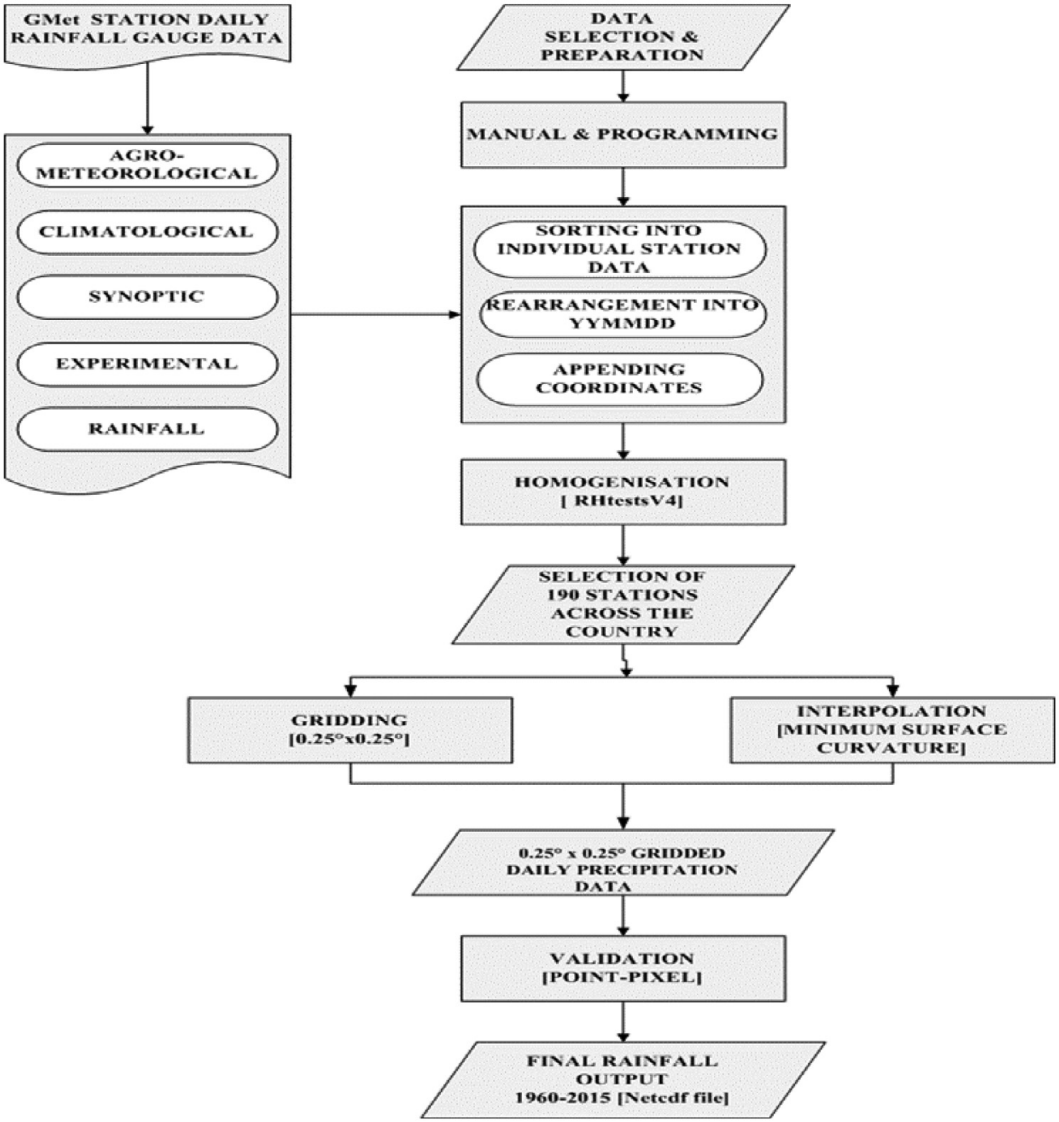
Data output approach.

**Fig. 2:**
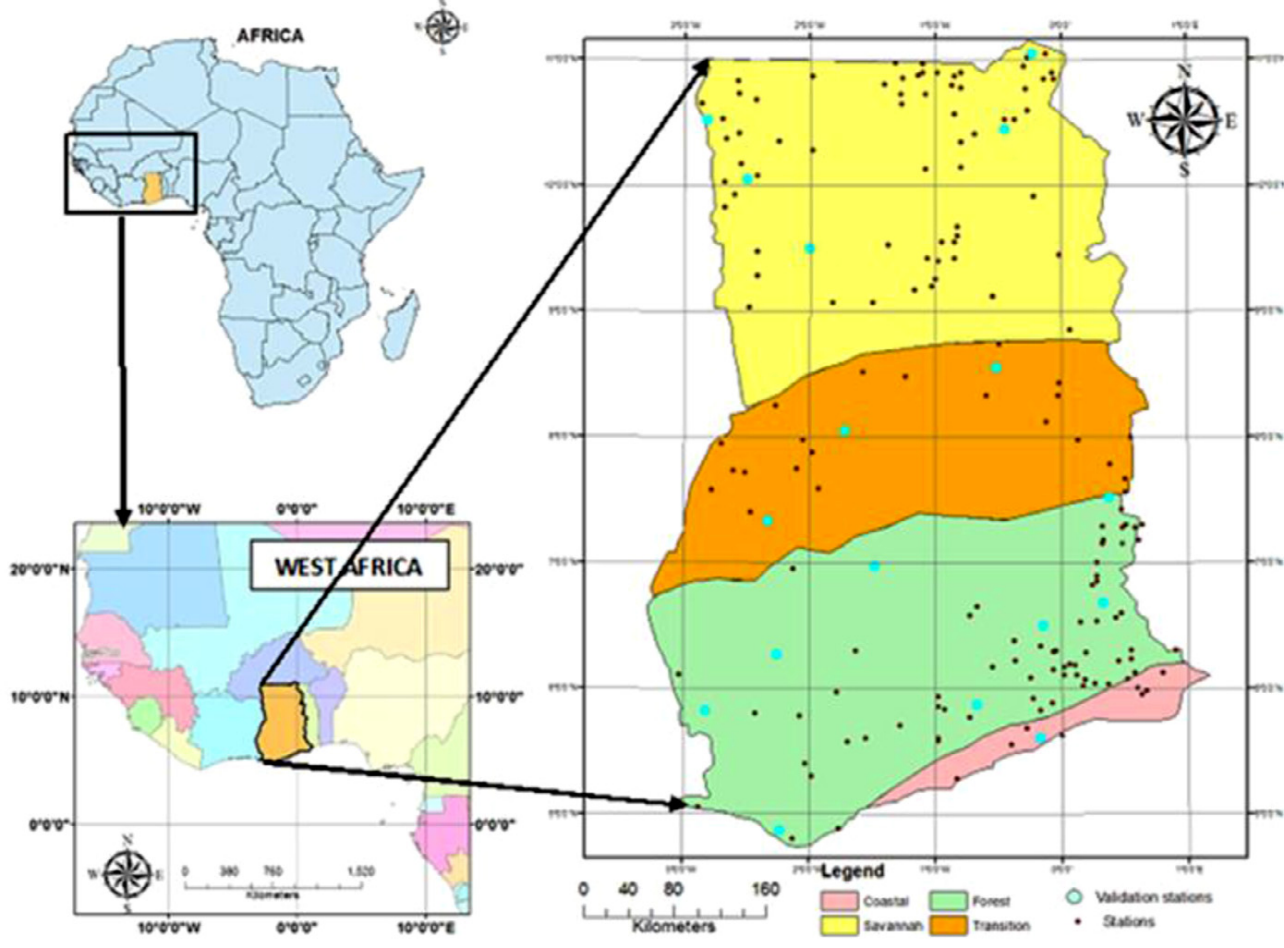
Map of Ghana showing the Agro-climatic Zones and location of gauge stations used.

**Table 1:** List of gauge stations from which secondary daily rainfall data was obtained.

Longitude	Latitude	Name of Gauge Station
-2.13	4.8	Princess Town
-2.23	4.87	Axim
-1.77	4.88	Takoradi
-2.88	5.05	HalfAssini
-0.98	5.93	AkimOda
-0.83	5.28	ApamSecSch
-1.98	5.3	Tarkwa
-2.03	5.4	Bogoso
-0.4	5.55	Weija
-1.7	5.57	Atieku
-0.98	5.58	BremanAsikuma
-0.17	5.6	Accra_KIAMO
-0.98	5.6	Nyakrom
-1.55	5.6	TwifoPraso
0	5.62	Tema
-0.28	5.68	Pokuase
-0.73	5.77	AkomAkroso
-2.08	5.78	WassaAkropong
-2.43	5.8	Asankragua
-0.17	5.82	Nsawam
-1.63	6.3	AkrokerriCollege
-2.82	5.82	Enchi
-0.93	5.83	Achiase
-0.98	5.85	AburiGardens
-0.67	5.87	Asamankese
-0.08	5.88	NunguaARS
-0.23	5.92	Pomadze
-2.26	6.27	Bibiani
0.28	6.53	Tsito
-0.98	5.93	AkimOda
-1.78	5.97	DunkwaOnOffin
-0.67	6.65	KwahuTafo
0.6	6	Sogakofe
0.37	6.03	Aveyime
0.52	6.07	Adidome
0.18	6.07	AsutuareFAO
0.52	6.07	Adidome
0.18	6.07	AsutuareSPC
-0.25	6.08	Koforidua
0.02	6.1	Somanya
0.12	6.1	AkuseNew
0.12	6.1	Akuse
0.02	6.1	SomanyaNew
0.8	6.12	Akatsi
0.58	6.12	Amelorkorpe
-2.47	7.4	Nsoatre
-0.07	6.15	KpongWaterworks
-0.07	6.15	KpongARS
-0.55	6.17	Kibi
-1.93	7.59	Techiman
0.5	7.56	Menuso
0.1	6.18	Ogoli
-0.37	6.22	AkimTafo
0.45	6.22	Workpe
0.55	6.23	KpedzegloMafi
0.55	6.3	AhundaAdoklu
-0.05	6.3	Akosombo
-0.17	6.33	Frankadua
-0.38	6.38	Begoro
-0.15	6.5	Anum
0.28	6.53	Tsitoclimate
0.15	6.53	AsantekromDodi
-0.73	6.58	Mpraeso
0.47	6.6	HoMet
0.33	6.68	Kpeve
0.28	6.85	Anfoega
-1.48	6.97	Jamasi
0.28	7	KpandoBHSS
0.48	7.15	Hohoe
0.48	7.28	AkpafuTodzi
-2.33	7.33	SunyaniMeteo
-2.33	7.33	SunyaniForestry
0.47	7.42	Jasikan
0.37	7.52	Worawora
-2.78	7.58	Gyapekrom
0.5	7.67	PapaswiDodi
-2.1	7.75	Wenchi
0.38	7.78	Adumadum
-1.98	7.88	Nchira
-2.7	7.95	Sampa
-2.05	7.98	BranamCamp
0.55	8.00	Breniase
-2.27	8.25	Bui
-0.6	8.33	Mankango
-0.03	8.33	Katiejeli
-0.02	8.43	Kpandae
-1.58	8.52	Buipe
-0.52	8.55	Salaga
0.07	8.85	Bimbila
-2.48	9.03	Bole
-1.17	9.17	Yapei
-1.82	9.07	Damango
-2	9.50	Mole
-1.5	9.07	Busunu
-1.03	9.20	Kusawgu
-2.42	9.28	Sawla
-0.98	9.40	Nyankpala
-0.85	9.42	TamaleMinistries
-1.07	9.42	Tolon
-0.02	9.45	Yendi
-2.42	9.48	Tuna
-1.38	9.53	Daboya
-0.95	9.55	Kumbungu
-0.85	9.55	TamaleAirNew
-0.83	9.60	Savelugu
-0.83	9.67	PongTamale
-2.68	9.83	Wechiau
-0.22	9.92	Gushiegu
-2.6	9.93	Vere
-2.68	10.03	Dorimon
-2.5	10.05	Wa
-2.42	10.08	Busa
-1.08	10.13	Kundungu
-0.8	10.15	Nasia
-2.55	10.17	Kaleo
-1.98	10.28	Funsi
-0.8	10.35	Walewale
-2.25	10.35	Kojopere
-2.67	10.37	Nadowli
-2.57	10.42	Dafiema
-0.45	10.45	Fumbisi
-2.82	10.52	Babile
-0.45	10.53	Gambaga
-0.37	10.53	Nalerigu
-2.7	10.53	Jirapa
-0.85	10.57	Pwalugu
-1.27	10.65	Wiaga
-2.87	10.65	Lawra
-2.43	10.68	Han
-1.28	10.73	Sandema
-2.57	10.73	NandomKo
-1.08	10.73	Kologu
-0.8	10.78	Zuarungu
-0.87	10.80	Bolgatanga
-0.87	10.80	Bolgatanga Agro
-2.58	10.83	Samoa
-0.14	10.85	Garu
-0.85	10.87	Vea
-1.98	10.87	Tumu
-0.8	10.9	Bongo
-0.07	10.9	Wuriyanga
-1.1	10.9	Navrongo
-0.3	10.95	Binduri
-1.1	10.97	Paga
-1.32	10.98	Kayoro
-0.27	11.02	Manga
-0.23	11.05	Bawku
0.12	11.05	Pusiga
-1.28	5.7	Fosu
-2.82	10.52	Babile
-1.72	8.05	Kintampo
0.43	6.56	Abutia
0.63	5.95	Agormeda
-0.07	6.29	NewAjena
-2.13	6.95	Amankwakrom
-2.61	7.73	Asuakaw
0.64	7.3	Baglo
0.02	6.17	BanaHill
-1.24	8.48	Banda
-1.26	10.86	Chiana
-0.12	8.12	Chinderi
0.67	5.98	Dabala
-3.03	6.11	Dadieso
-0.5	8.74	Dagonkade
0.52	6.07	AdidomeDuffor
-0.69	10.41	Guabuliga
-0.28	10.77	Kugri
0.32	7.29	Kwamikrom
0.61	7.18	Likpe
0.28	6.89	MafiAnfoe
0.9	6.3	MatseHave
-0.99	10.9	Mirigu
-1.41	10.81	Nakong
-0.06	10.85	Nangodi
0.58	7.28	NewAyoma
0.32	7.15	NkonyaAhenkro
0.17	6.02	Osudoku
-0.55	9.12	Palbe
0.19	6.28	Pore
-0.27	10.6	Sakogu
-1	9.26	Sankpala
-2.51	7.72	Seikwa
0.06	6.19	Senchi
0.13	7.98	Sokode
-2.31	7.33	Sunyani
0.51	7.3	Teteman
-1.14	10.88	Tono
0.33	7.18	Wurupong
0.24	6.82	Wusuta
0.26	6.04	Kasunya

**Table 2:** Distribution of Gauge stations in the four (4) zones.

s/n	Agro-climatological zone	Size (km²)	Percentage (%)	No. of stations	Percentage (%)
1	Northern Savannah	87,584	37	70	37
2	Forest transition	57,566	24	27	14
3	Forest	84,897	36	80	42
4	Coastal	8,488	4	13	7
	Total	238,535	100	190	100

**Fig. 3:**
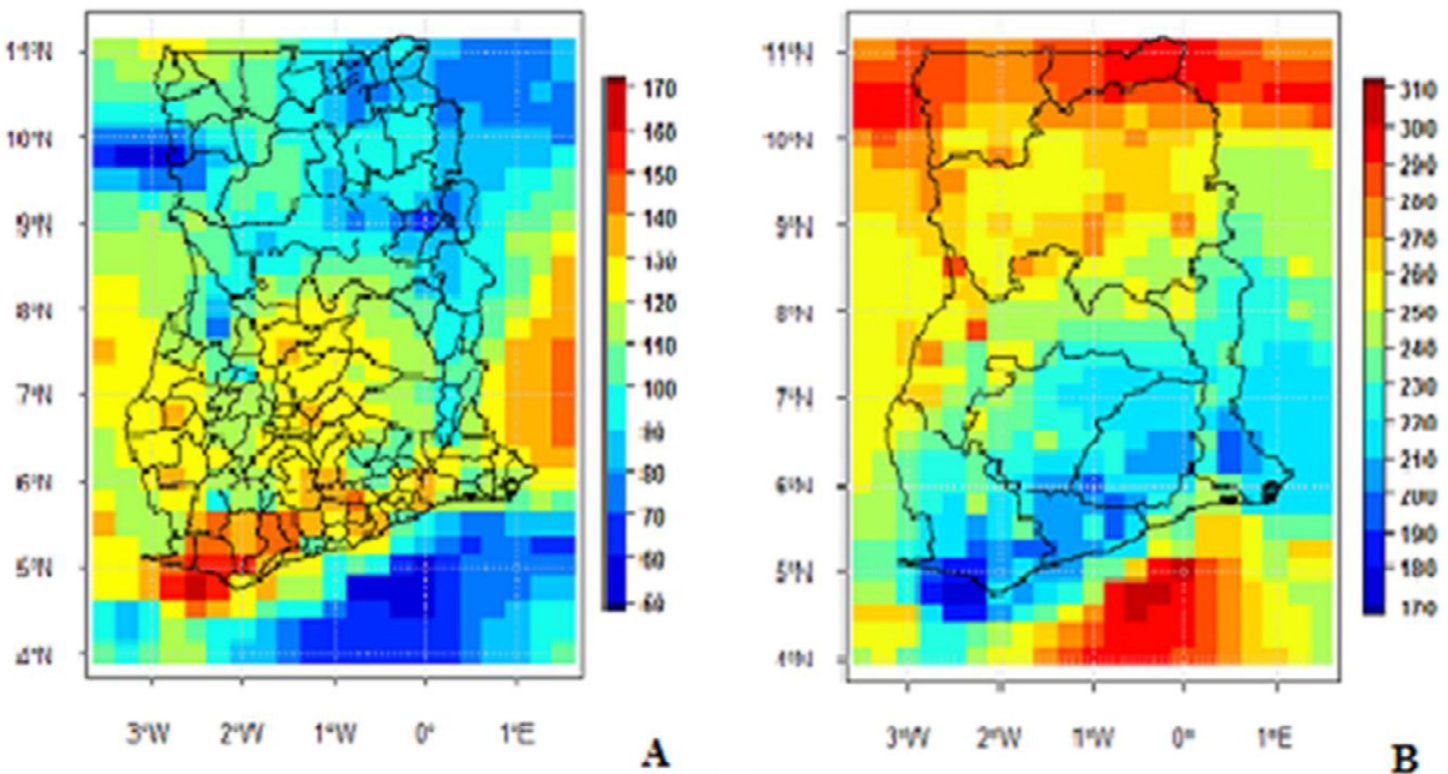
Map showing mean Number of Wet Days (A) and Dry Days (B) (1960-2015).

**Fig. 4:**
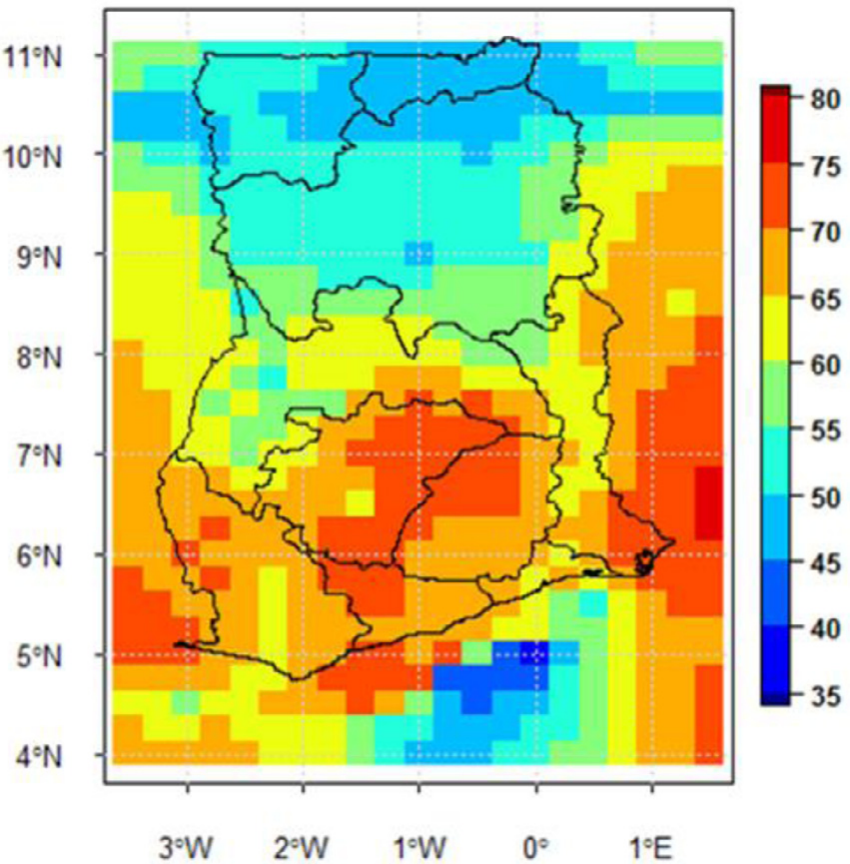
Map showing mean Number of Wet Spells 1960-2015.

## 3 Experimental Design, Materials and Methods

This data is the output from an interpolation of daily rainfall gauge data for 190 stations across all the four(4) agro-ecological zones of Ghana. It relied on available data from all the stations, though a consistent flaw in the input data was the many years in which there was no data recorded for some stations. This inadequacy is the reason for the interpolation for 56-year period (20,454 days) covering the entirety of the country Ghana.

The process for the generation of the new dataset involved using python scripts for the interpolation (gridding). The specific python scripts used are provided are listed below and can be obtained from; https://github.com/Ampofo16693/Daily-Rainfall-Gridding

• Merging Individual Excel file for all 190 stations and interpolation
• Assigning Grid
• Replacing negative values using python

Data gridding was performed using Minimum Surface Curvature with tensioning as proposed [6]. The point data were first pre-processed with ‘blockmean’, per every 0.25*°*  ×  0.25*°* spatial grid, to avoid spatial aliasing and remove redundant data [1]. This step allowed to average datasets per defined grids, maintaining spatial signatures within each grid. A tension factor of 1 was used in gridding, which works to suppress undesired oscillations and false maxima/minima seldom generated by MSC. The MSC with set tension was iterated on the daily data till convergence was met, which is e^−4^ of the root mean square deviation of the data from a best-fit plane.

In place of the error fields, we provide the results of an accuracy assessment and validation which was done using 19 gauge station results to test the accuracy of the output data at these specific locations. A summary of these results for the seasons is computed for the ratio and correlation of the observed gauge data to the output is provided in Tables 3 and 4.

**Table 3:** Ratio of the 19 observed station data to Gridded data.

Station	Spring	Summer	Autumn	Winter	Average
Accra_KIAMO	1.1479	0.6531	0.6132	1.5606	**0.9937**
Anum	0.5353	0.4893	0.8978	0.7572	**0.6699**
Axim	0.6911	0.3470	0.2593	0.6677	**0.4913**
Wa	0.7106	0.9581	1.3571	0.9346	**0.9901**
Mole	4.3131	0.7340	0.7961	1.3769	**1.8050**
Bawku	2.8231	1.4628	2.4788	1.4752	**2.0600**
Enchi	0.5264	0.6701	0.6628	0.9581	**0.7043**
Kpeve	0.4193	0.6826	1.0192	1.0556	**0.7942**
Babile	1.1391	1.3750	2.7886	1.8356	**1.7846**
Jamasi	1.2753	0.8479	0.9742	0.9508	**1.0121**
Salaga	0.9902	0.6910	0.7042	0.9021	**0.8219**
Bibiani	0.7534	0.7485	0.8004	1.1375	**0.8599**
Bimbila	0.6871	0.8848	0.5912	0.8504	**0.7534**
Fumbisi	0.1697	0.7058	2.0219	1.4167	**1.0785**
Mpraeso	1.3854	0.7068	1.0348	0.8144	**0.9853**
Kintampo	0.0021	0.6097	0.6272	0.7967	**0.5089**
Worawora	0.6450	0.7179	0.4899	1.1443	**0.7493**
Asamankese	0.6406	0.5565	0.8893	0.8508	**0.7343**
SunyaniMeteo	0.4773	0.7693	0.7817	1.0328	**0.7653**
**Average**	**1.0175**	**0.7690**	**1.0415**	**1.0799**

**Table 4:** Correlation between the 19 observed station data to Gridded data.

Station	Spring	Summer	Autumn	Winter	Average
Accra_KIAMO	0.5565	0.7445	0.4320	0.5607	**0.5734**
Anum	0.7571	0.5673	0.7378	0.4927	**0.6387**
Axim	0.5349	0.6607	0.6536	0.6692	**0.6296**
Wa	0.1318	0.5338	0.6002	0.4266	**0.4231**
Mole	0.5351	0.6420	0.7922	0.5731	**0.6356**
Bawku	0.8460	0.3464	0.6578	0.7590	**0.6523**
Enchi	0.6196	0.8761	0.6739	0.8032	**0.7432**
Kpeve	0.4196	0.4584	0.4650	0.4027	**0.4364**
Babile	0.0195	0.1120	0.1550	0.7474	**0.2487**
Jamasi	0.6210	0.6940	0.6915	0.6354	**0.6605**
Salaga	0.7211	0.8740	0.7434	0.6462	**0.7462**
Bibiani	0.6508	0.8100	0.7239	0.8352	**0.7550**
Bimbila	0.4217	0.6097	0.7793	0.6387	**0.6124**
Fumbisi	0.5369	0.0053	0.5935	0.8150	**0.4850**
Mpraeso	0.3456	0.6696	0.5651	0.5521	**0.5331**
Kintampo	0.7462	0.8544	0.7066	0.0669	**0.5935**
Worawora	0.4940	0.7050	0.5341	0.6254	**0.5896**
Asamankese	0.4352	0.6964	0.4624	0.6445	**0.5596**
SunyaniMeteo	0.5385	0.7580	0.7988	0.8375	**0.7332**
**Average**	**0.5206**	**0.6109**	**0.6193**	**0.6175**	

## Ethics Statements

The work outlined above did not involve human subjects for which relevant informed consent was required.

There were no animal experiments, so compliance with any guidelines was irrelevant.

Primary data used for this work was not obtained from any social media platform but empirical data obtained from a public institution.

## CRediT Author Statement

**Steve Ampofo:** Writing – Original draft preparation, Methodology, Data Curation; **Thompson Annor:** Conceptualisation, Supervision, Editing; **Jeffrey N. A. Aryee:** Data curation, Software application, Validation; **Jacob Agyekum:** Data curation; **Leonard K. Amekudzi:** Conceptualisation, Supervision, Reviewing & Editing.

## Declaration of Competing Interest

The authors declare that they have no known competing financial interests or personal relationships that could have appeared to influence the work reported in this paper.

## Data Availability

Gridded Daily Rainfall Data for Ghana for the period 1960 - 2015: Approach and Validation Process (Original data) (Mendeley Data).
